# Supplementary material for: Chromothripsis during telomere crisis is independent of NHEJ, and consistent with a replicative origin
Source: Genome Res. 2019 May;29(5):737–49. doi: 10.1101/gr.240705.118 (PMC6499312; doi:10.1101/gr.240705.118)
Supplement: Supplemental Material [file supp_gr.240705.118_Supplemental_file_1.zip › contigs/annotated_contigs/DB116/contig.2.DB116_length_427_mean_cov_5.26932084309.docx]

**DB116_length_427_mean_cov_5.26932084309**

```
ATGCTCATTCTCTTTGGACCCAGTGTTTCCTTACTAGGGATGGAAATTAGGGGTGGAGTGATGGAGTGGGAGGGGGTGAATTGTATGAT
 >chr9:23004466-23004682 + E=2e-118
CTAAGGTGTTATCCTCTCCTCTGATTCCTACACCTCATGCATATTTGGAAGCCGCAGAAACATTGTTGTGGCCCCTGAGTTGTTCTACT

TGCCCTATGAAAGTGGTAGGCTTCACACAGACAACAAT|C|TTGATGGTCTGTAAATATAAGAGGTGTGTTCTTCCCTCTTTTCTTTCT
                                        >chr9:23008691-23008901 + E=7e-106
AAGTGTAACAATTGATTAAAAATGAAAATGCATGCTTTTCAATTGAAATGAATGTTATCAACACAAATATCACTCCAGATACAGCTCAA

TCGAATCAAGCCAAAATAAATGTTGCCAAACTAAATTGTTTCATGTGTTTTCTGGTCTCCTTTCATATGTCTA
```
